# Innate Viral Sensor MDA5 and Coxsackievirus Interplay in Type 1 Diabetes Development

**DOI:** 10.3390/microorganisms8070993

**Published:** 2020-07-03

**Authors:** Samuel I. Blum, Hubert M. Tse

**Affiliations:** Department of Microbiology, Comprehensive Diabetes Center, The University of Alabama at Birmingham, Birmingham, AL 35294, USA; sblum@uab.edu

**Keywords:** type 1 diabetes, melanoma differentiation-associated protein 5 (MDA5), coxsackievirus type B, type I interferons, IFIH1

## Abstract

Type 1 diabetes (T1D) is a polygenic autoimmune disease characterized by immune-mediated destruction of insulin-producing β-cells. The concordance rate for T1D in monozygotic twins is ≈30–50%, indicating that environmental factors also play a role in T1D development. Previous studies have demonstrated that enterovirus infections such as coxsackievirus type B (CVB) are associated with triggering T1D. Prior to autoantibody development in T1D, viral RNA and antibodies against CVB can be detected within the blood, stool, and pancreata. An innate pathogen recognition receptor, melanoma differentiation-associated protein 5 (MDA5), which is encoded by the *IFIH1* gene, has been associated with T1D onset. It is unclear how single nucleotide polymorphisms in *IFIH1* alter the structure and function of MDA5 that may lead to exacerbated antiviral responses contributing to increased T1D-susceptibility. Binding of viral dsRNA via MDA5 induces synthesis of antiviral proteins such as interferon-alpha and -beta (IFN-α/β). Viral infection and subsequent IFN-α/β synthesis can lead to ER stress within insulin-producing β-cells causing neo-epitope generation, activation of β-cell-specific autoreactive T cells, and β-cell destruction. Therefore, an interplay between genetics, enteroviral infections, and antiviral responses may be critical for T1D development.

## 1. Introduction

Type 1 diabetes (T1D) is a chronic inflammatory autoimmune disease where pancreatic β-cells are destroyed by autoreactive T cells [1,2]. Currently, ≈1.5 million people are living with T1D in the United States [3]. T1D incidence is expected to increase by ≈2–5% annually worldwide [4,5] resulting in ≈128,900 new cases [6]. While T1D can be treated by exogenous insulin injections, there are still major comorbidities that are associated including cardiovascular disease, nephropathy, neuropathy, and retinopathy [4]. While genetic susceptibility is required for disease, ≈30–50% concordance rates of T1D in monozygotic twins suggest that genetics alone cannot cause disease and that environmental factors likely contribute to disease induction [7,8,9,10,11].

The highest genetic risk for T1D is conferred by the HLA class II haplotypes HLA-DR3-DQ2 and HLA-DR4-DQ8 as well as mutations in the *INS* (insulin) gene [12]. However, single nucleotide polymorphisms (SNPs) in immune response genes such as *DDX58* (DExD/H-Box Helicase 58), *TLR2* (Toll-like receptor 2), *TLR3* (Toll-like receptor 3), *TLR7* (Toll-like receptor 7), *TYK2* (tyrosine kinase 2), and *IFIH1* (Interferon Induced With Helicase C Domain 1) are implicated with T1D disease risk [13,14,15,16,17,18,19,20,21,22,23,24,25]. Mutations in these innate sensors provide a key link between genetics and the environment for T1D initiation.

Enteroviral infection may be a key environmental factor that contributes to the development of T1D by inducing a robust antiviral response [26,27]. An enterovirus associated with T1D is coxsackievirus (CVB) [28]. There are two groups of coxsackievirus, A and B, which both cause hand, foot, and mouth disease (HFMD) [29,30]. The coxsackievirus type A (CVA) group has 24 serotypes; this group causes flaccid paralysis and severe infection [31,32]. The coxsackievirus type B (CVB) group has six serotypes, all correlated with T1D, known for causing spastic paralysis with mild infections [33,34,35,36,37,38,39,40,41,42,43,44,45]. CVB infection is transmitted by the fecal–oral route or by direct contact with mucosal secretions. CVB initially replicates within the gut, spleen, and then disseminating to the heart, central nervous system (CNS), and pancreata [31,45,46,47,48].

CVB has been shown to infect cardiomyocytes and pancreatic cells, inducing myocarditis and pancreatitis in both mice and humans [48,49,50,51,52]. Yet, differences do exist when CVB infects mouse and human pancreatic cells. CVB can readily infect mouse pancreatic exocrine cells, but poorly infects mouse endocrine cells. Conversely, CVB can infect human endocrine cells, but human exocrine cells are less susceptible to infection [43,53,54,55,56,57]. While it remains unclear if CVB infection can directly result in the catastrophic loss of β-cells in human T1D, a robust proinflammatory antiviral response in T1D genetically-susceptible individuals may initiate β-cell destruction. Several mechanisms have been proposed to explain how CVB may trigger T1D development including β-cell death, bystander activation of autoreactive T cells, and destruction of pancreatic cells [58,59]. Regardless of the proposed mechanism, viral sensing and antiviral responses are critical for the development of CVB induced T1D.

A sensor of CVB infections is the RNA helicase enzyme melanoma differentiation-associated protein 5 (MDA5), a RIG-I-like receptor encoded by the *IFIH1* gene, that senses double-stranded RNA (dsRNA) and has been associated with T1D [60,61]. Stimulation of MDA5 leads to the synthesis of type I interferons (IFN-α/β) to restrict viral replication and to activate the immune system [62]. Production of IFN-α/β and activation of the immune system following CVB infection may be the main driving force for the onset of T1D in genetically-susceptible individuals. This review article will discuss the collective contributions of CVB infections, the innate sensor MDA5, IFN-α/β, antiviral responses from pancreatic β-cells, and the immune system in T1D development.

## 2. Enteroviruses and their Association with T1D

For decades, viral infections were suggested to induce T1D by activating autoimmune lymphocytes that cause direct β-cell destruction [63,64,65]. Viruses such as CVB, echovirus, rotavirus, mumps, rubella, cytomegalovirus, and endogenous retroviruses (ERVs) have been proposed to cause islet autoimmunity, indicating antiviral responses may trigger T1D [42,44,66,67,68,69,70,71,72,73,74,75,76,77]. ERVs have been identified in both Non-Obese Diabetic (NOD) mice, a spontaneous mouse model of T1D, and human T1D islets [76,78,79,80]. Presentation of ERV antigens including Gag has been associated with T1D induction, by activating autoreactive T cells and the generation of IFN-γ [76,80]. Children are exposed to many pathogens early in life, including viral infections that are associated with triggering T1D [81,82].

Children that develop T1D and have autoantibody seroconversion, experience more infections earlier in life than non-diabetic, HLA-matched controls [83]. Children with viral respiratory infections in the first 6 months of life have a significant increased risk of T1D development compared to children without any viral respiratory tract infections [84]. The Diabetes Auto Immunity Study in the Young (DAISY) study examined illnesses in the first 9 months of life in individuals without a family history of T1D and individuals with a first-degree relative with T1D. Their findings showed an increased number of gastrointestinal illnesses associated with increased risk of T1D autoantibody development in children who consumed a gluten-based diet. They also concluded that pathogens such as enteroviruses may increase the risk of autoantibody development in the presence of an inflammatory diet [82]. A study in Taiwan reported that children infected with an enterovirus have a 50% higher chance of developing T1D one year-post infection compared to uninfected children [85].

In some reports, patients with T1D have detectable anti-enteroviral antibodies and CVB RNA in the blood and stool 6–12 months before autoantibody development [86,87]. Moreover, some studies have shown that recent onset patients with T1D have detectable enterovirus infections within their pancreata and islets [34,88,89,90]. Patients with fulminant T1D have robust expression of MDA5, RIG-I, and the major capsid protein of enteroviruses, VP1, in both β-cells and α-cells within the islets compared to long-standing diabetic patients and non-diabetic controls [22]. Furthermore, cellular infiltrates, possibly immune cells, within the pancreata of these patients had high expression of TLR3 and TLR4 [22], indicating the induction of a proinflammatory innate immune response to an infection.

The increased prevalence in T1D worldwide may be in part due to the increased prevalence of coxsackievirus infections. A study looking at the prevalence of HFMD in China discovered that the incidence of CVA-associated HFMD fell from 97% in 2010 to 82.6% in 2011. However, CVB-associated HFMD incidence increased from 3% in 2010 to 17.1% in 2011 [86,91], which may explain increased T1D incidence. Europe does not currently have a virus surveillance program in place, but does have widespread circulation of over 30 different enteroviruses including diabetes-associated viruses CVB and echovirus [92]. Fortunately, the Center for Disease Control and Prevention (CDC) has developed the national enterovirus surveillance system to monitor the detection of enteroviruses and parechoviruses in the United States [93]. In the future, long-term longitudinal studies may discover an increased prevalence of diabetogenic CVB infections within the United States and worldwide to parallel the increased frequency of T1D development.

The hygiene hypothesis may also account for the rise in T1D cases. This hypothesis suggests that reduced early-life exposure to a wide spectrum of pathogens may compromise immune system maturation and thereby perpetuate an increase in allergic and autoimmune responses. Early exposure to a diverse range of microbes is critical to train the immune system for efficient anti-microbial responses to foreign-antigens and not self-antigens. There is a clear negative correlation between sanitary conditions and T1D. The frequency of T1D is lower in underdeveloped areas with poor hygienic conditions [94,95], while developed countries have increased prevalence of T1D [96,97,98]. Furthermore, T1D incidence increases in children who have below normal levels of exposure to common pathogens early in life [99]. Experimental models utilizing NOD mice have further validated the hygiene hypothesis. Young NOD mice exposed to bacteria, viruses, and parasites can delay T1D progression [100,101,102,103,104]. Reduced exposure to common pathogens early in life may lead to strong antiviral responses to diabetogenic pathogens, like CVB when exposed later in life, resulting in IFN-α/β synthesis, β-cell destruction, and T1D. More research is needed to fully grasp the role of CVB infection in T1D development, including MDA5-dependent antiviral responses and the synthesis of IFN-α/β.

## 3. Type I Interferons in T1D

Type I interferons (IFN-α/β) are cytokines that impair early viral replication, regulate immune cell activation, and promote the production of proinflammatory cytokines [62,105]. IFN-α/β are generated following innate pattern-recognition receptor (PRR) interactions with microbial-associated molecular patterns (MAMPs) from viruses, bacteria, fungi, and parasites [106]. During infection with ssRNA viruses, key PRRs such as MDA5, RIG-I, and TLR3 will recognize dsRNA generated during viral replication. This results in the activation of a signaling cascade, including the phosphorylation of NF-κB, IRF-3, and IRF-7 to induce IFN-α/β synthesis [107].

IFN-α/β functions in both an autocrine and paracrine manner to promote the activation of immune cells, cytokine production, and interferon-stimulated gene transcription for viral clearance [108]. Unfortunately, aberrant synthesis of IFN-α/β can have deleterious effects on peripheral tolerance. Persistent synthesis of IFN-β has been shown to impair anti-inflammatory IL-10 signaling in NOD mice [109] and heightened IFN-α/β expression has been associated with T1D development in both mice and humans [110,111]. IFN-α/β not only alters immune cell responses but negatively impacts β-cell function. Heightened IFN-α/β synthesis impairs β-cell insulin production, proinsulin conversion, cellular replication, electron, and ATP transportation [112,113,114,115,116,117].

Prior to T1D onset, a type I interferon transcriptional gene signature is detected in peripheral blood mononuclear cells [118]. Patients with T1D have increased IFN-α levels within the pancreata and enhanced IFN-α synthesis from peripheral blood plasmacytoid dendritic cells compared to healthy patients [119,120]. Interferon therapy can rapidly accelerate T1D development in genetically-susceptible individuals [121], indicating IFN-α/β may be able to initiate T1D by activating autoreactive lymphocytes. Consequently, viral infections with high levels of IFN-α/β have been documented to dysregulate regulatory T cell (Treg) function [122]. However, complete ablation of IFN-α/β leads to a loss of FOXP3 expression and Treg function, which can be rescued with exogenous IFN-α [123]. There is a clear duality in the role of IFN-α/β. It is critical for response to infection, but loss of or excess IFN-α/β synthesis can destabilize peripheral tolerance subsequently leading to T1D development. While there are reports that a type I interferon signature is present in patients prior to the onset of T1D, this is not always evident and predictive of a direct correlation with autoimmunity. Analysis of IFN-α/β synthesis by neutrophils in the peripheral blood of patients with systemic lupus erythematosus (SLE) and healthy controls did not show a reliable biomarker of disease progression [124,125,126]. Therefore, more research is needed to fully understand the association between IFN-α/β synthesis and autoimmunity.

Studies investigating the role of IFN-α/β in initiating T1D in NOD mice found that NOD mice have an IFN-α gene signature prior to T1D onset similar to human T1D patients [127]. Additionally, suppressing IFN signaling through neutralizing antibodies to the interferon-alpha and -beta receptor subunit 1 (IFNAR1) was effective in significantly delaying T1D onset [127,128]. Paralleling the neutralization of IFNAR1 to delay T1D progression, IFNAR1^KO^ mice also have a significant delay in T1D development [129,130]. Transgenic mice where β-cells constitutively express IFN-α or IFN-β rapidly develop T1D, indicating that high expression of IFN-α/β can break peripheral tolerance [130,131]. However, following LCMV infection blockade of IFN-α impairs lymphocyte tracking into islets and T1D, whereas blocking IFN-β elicits no protective diabetic effects in mice [132]. This may be due to the fact IFN-β has been shown to impair proliferation, while IFN-α has a stronger proinflammatory antiviral effect [133,134,135]. However, more studies are required to fully understand the roles of IFN-α/β in T1D development. Unfortunately, blocking IFN-α/β activity is not a possible solution, as they have very important functions within the immune system that are critical for survival. Therefore, selective targeting of the IFN pathway may provide a therapeutic benefit by reducing, but not completely impairing IFN-α/β synthesis and IFN-α/β-dependent responses.

## 4. Pancreatic β-Cell Response to Viral Infection in T1D

Enteroviral infection of pancreatic β-cells may be a driving force for T1D development. β-cells like other nucleated cells can generate antiviral responses, synthesize IFN-α/β, promote the activation of autoreactive T cells, and initiate T1D [37,136,137,138]. Human and mouse β-cells express varying levels of Coxsackievirus and Adenovirus Receptor (CAR), which allows for cellular infection and replication of the virus [139,140,141]. There are five known isoforms of CAR that have been shown to bind enteroviruses and allow for cell entry, CAR2/7 CAR3/7, CAR4/7, CAR^EX7^, and CAR^EX8^ [142,143]. Human β-cells express two isoforms of CAR called CAR^EX7^ and CAR^EX8^. CAR^EX7^ is encoded by the 1st seven exons of the *CXADR* gene, and CAR^EX8^ is created by a cryptic splice site within the 7th exon of *CXADR* [144]. CAR^EX7^ has been detected in β-cells, but not in any other exocrine pancreatic cells, while CAR^EX8^ is proposed to be expressed in exocrine pancreatic cells [144]. Kotha et al. demonstrated that apical localization of CAR^EX8^ in the epithelial airway of the lung is required for the initiation of adenovirus infections in the lung [145]. Therefore, diverse CAR isoforms may provide unique cell specificity for different viral infections in many sites throughout the body. C57BL/6J mice lacking CAR expression on pancreatic cells are protected from pancreatic CVB infection and pancreatitis [146], and patients with T1D display an increase in CAR expression within the pancreata, compared to non-diabetic patients [147]. Therefore, high CAR expression levels on β-cells may promote the initiation of T1D due to CVB infection. Unfortunately, the association between specific isoforms of CAR for CVB-induced T1D is not understood. It is plausible that the unique isoform of CAR^EX7^ found on β-cells may have a critical role in CVB infection and the initiation of T1D in genetically-susceptible individuals.

**Figure 1:**
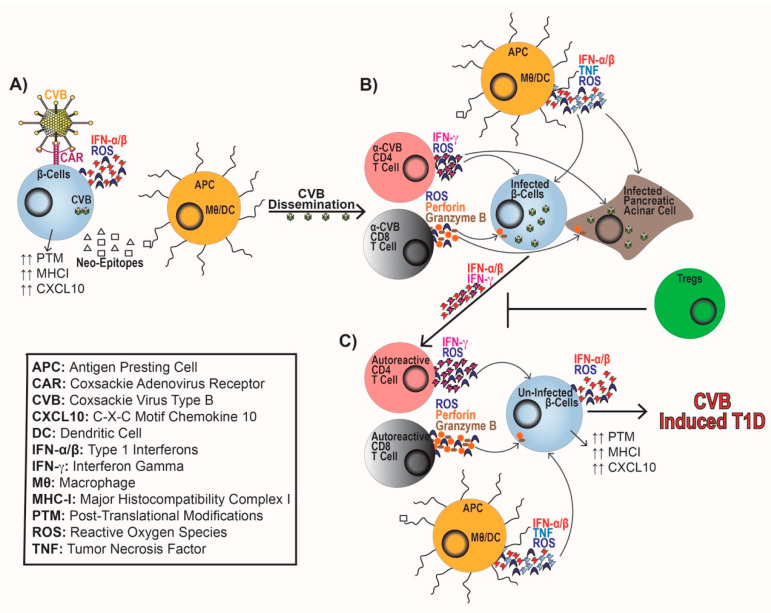
Model of coxsackievirus type B (CVB)-induced type 1 diabetes (T1D). (**A**) Coxsackievirus binds to the Coxsackievirus and Adenovirus Receptor (CAR) on the β-cell leading to β-cell infection, cellular dysregulation, interferon-alpha and -beta (IFN-α/β) synthesis, and generation of neo-epitopes. Phagocytosis and presentation of neo-epitopes by antigen-presenting cells (APCs) leads to the activation of autoreactive CD4 and CD8 T cells. (**B**) CVB dissemination leads to the generation and differentiation of CVB-specific CD4 and CD8 T cells. Both CD4 and CD8 T cells will gain effector responses, CD4 T cells will produce ROS and IFN-γ. Whereas, CD8 T cells will produce ROS, perforin, and granzyme B targeting infected β-cells and acinar cells, while APCs will concomitantly produce ROS, IFN-α/β, and TNF to promote viral clearance and immune cell activation. (**C**) The proinflammatory response to CVB infection and generation of IFN-α/β and IFN-γ leads to the loss of Treg peripheral tolerance. Subsequent activation of proinflammatory APCs and autoreactive CD4 and CD8 T cells further propagate CVB-induced T1D.

ERVs and persistent CVB infection of β-cells can induce endoplasmic reticulum (ER) stress, IFN-α/β synthesis, and the upregulation of Class I HLA and CXCL10 expression to recruit and activate immune cells [76,78,79,148,149,150]. ER stress on a β-cell can perpetuate T1D progression by causing mitochondrial dysfunction, reduced insulin production, synthesis of reactive oxygen species (ROS), and induction of cell death pathways [148,151,152,153,154,155]. ER stress can also elicit protein misfolding and post-translational modifications leading to neo-antigen generation by β-cells. These novel β-cell antigens can facilitate epitope spreading and be presented by antigen-presenting cells (APCs), such as macrophages and dendritic cells to activate naïve autoreactive T cells [148,153,156] (Figure 1A).

Not only can viral infections cause β-cell-mediated ER stress, but CVB infection can also dysregulate the synthesis of β-cell-derived micro RNAs (miRNAs) [157]. CVB infection of human pancreatic islets induces the generation of ≈33 miRNAs that are predicted to directly target over 50 genes associated with T1D [157], including the upregulation of miR-432, miR-101a, miR-30b, and miR-342. Increased expression of miR-432, miR-101a, and miR-30b can increase viral replication, reduce insulin production, and enhance cytokine-mediated β-cell dysfunction, respectively [158,159,160]. CVB infection can also increase miR-342 synthesis, which directly interacts with the mRNA silencer of islet autoantigen IA-2β, leading to increased levels of autoantigens [161]. Increased expression of autoantigen IA-2β following CVB infection may be a driving factor in T1D development by activating autoreactive T cells.

Pancreatic α-cells have faster kinetic antiviral response than β-cells within the first 8 h of CVB infection [162]. This may hint that β-cells may not produce a robust antiviral response to control viral infections. Rat β-cells infected with CVB undergo apoptosis 48 h post-infection, while infected α-cells are resistant. However, α-cells and β-cells stimulated with cytokines and poly(I:C) have similar death rates [162]. CVB infection can affect pancreatic β-cells survival by causing necrosis, apoptosis, and T cell-mediated destruction [59]. Transplantation of human islets into streptozotocin-induced diabetic mice devoid of T and B cells can restore blood glucose to euglycemic levels. However, following infection with CVB, 50% of the mice engrafted with the human islets were diabetic at 28 days post-infection; whereas, uninfected mice remained euglycemic [140]. This suggests that CVB may be directly damaging β-cells. CVB infection can activate proinflammatory innate immune responses to mediate β-cell destruction causing the loss of the transplanted human β-cells and T1D. Overall, the poor antiviral response of β-cells may increase the susceptibility to infection, apoptosis, and increased antigen presentation of β-cell epitopes to activate autoreactive T cells.

## 5. Immune Cell Response to Viral Infections in T1D

In addition to direct damage to β-cells, CVB infection results in the activation of immune cells. During CVB infection, MDA5 recognizes dsRNA intermediates of CVB that leads to the production of IFN-α/β, chemokines (CCL2, CCL5, and CXCL10), and upregulation of Class I HLA [44,149,163]. This response leads to the activation and trafficking of both the innate and adaptive arms of the immune system. Chemokine synthesis leads to the recruitment of APCs, which upregulate MHC-II. APCs generate IL-6, tumor necrosis factor (TNF), and ROS [164] as an antiviral response, while concomitantly regulating T cell entry into islets allowing for β-cell destruction [128,165,166] (Figure 1B). APCs promote the activation of CD4 to produce IFN-γ and ROS to augment antiviral responses. Whereas, CD8 T cells produce ROS, perforin, and granzyme B to promote viral clearance (Figure 1B). This proinflammatory response can cause bystander activation of β-cell-specific T cells [166,167,168,169,170,171,172,173,174] (Figure 1C). If peripheral tolerance mechanisms including Treg cells are not able to effectively inhibit autoreactive T cell responses, T1D progression ensues [175].

Tregs play a critical role in maintaining peripheral tolerance and preventing the development of autoimmunity [176,177]. Patients with T1D experience loss of Treg differentiation and function furthering disease progression [178,179], including reduced IL-2 sensitivity, unstable FOXP3 expression, and increased apoptosis [180,181,182,183,184]. Patients with T1D also experience miR-21, miR-31, and miR-146 dysregulation causing altered FOXP3 expression and impaired Treg function [185,186,187,188]. Adoptive transfer of Tregs in NOD mice delayed spontaneous T1D development and CVB-accelerated T1D, indicating that Tregs are required to prevent T1D. The augmented Treg population restored peripheral tolerance and prevented β-cell damage both spontaneously and following CVB infection without compromising viral clearance [189,190,191]. Following viral infections, Treg populations are dramatically reduced, and a proinflammatory Th1 cytokine response is generated [192]. This reduction in Treg cells may promote a heightened antiviral response. CVB infection may lead to a loss of peripheral tolerance, initiate T cell autoimmunity, and perpetuate T1D onset (Figure 1C).

## 6. Function of MDA5

MDA5 was first discovered in 2001 as an IFN-β-inducible protein with dsRNA binding, ATPase activity, and the ability to suppress human melanoma cell growth by regulating interferon-mediated cell growth and apoptosis [193,194]. MDA5 recognizes positive-sense single-stranded non-enveloped RNA viruses, such as enteroviruses and noroviruses, and prevents early replication of CVB infection [163,195,196,197,198,199]. Additional reports showed that MDA5 preferentially binds long dsRNA greater than 1000 bp with no 5′ cap and can induce IFN-α/β synthesis [200,201,202]. Multiple SNPs in *IFIH1* have been associated with risk or protection for T1D, potentially due to modification of the structure and function of MDA5 [14].

**Figure 2:**
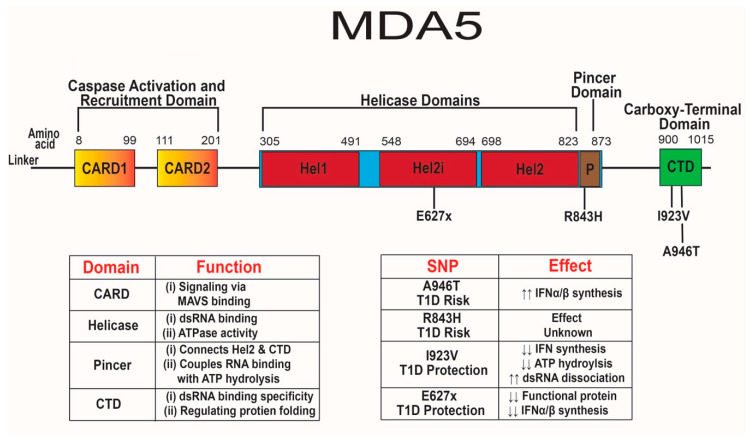
Diagram of melanoma differentiation-associated protein 5 (MDA5) functional domains and *IFIH1* single nucleotide polymorphisms (SNPs). ↑↑ indicates increased functionality, whereas ↓↓ indicates decreased functionality. The N-terminal CARD domains of MDA5 are located between amino acid residues 8–201, with no known SNPs in MDA5 associated with T1D. The helicase domains in MDA5 are located between residues 305–873, containing helicase sub-domains Hel1, Hel2i, Hel2, and a pincer domain. The Hel1 subdomain comprises of amino acid residues 305–491, Hel2i subdomain contains amino acids 548–694, and the Hel2 subdomain is located at amino acid positions 698–823. The T1D protective E627x SNP is located within the Hel2i subdomain. The pincer domain is located within the main helicase domain of residues 824–873 and contains the R843H SNP associated with T1D susceptibility. Finally, the C-terminal domain (CTD) located between residues 900–1015 contains both SNPS I923V and A946T associated with both T1D protection and risk, respectively.

MDA5 has two N-terminal caspase activation and recruitment domains (CARD), three separate helicase domains, a pincer domain, and a C-terminal domain (CTD) [60,203] (Figure 2). The pincer domain forms a closed ring around RNA, while also linking the helicase domain and the CTD [203]. Both the helicase domains and CTD promote binding to the stem of dsRNA [60,204,205]. Dephosphorylation of serine 88 by phosphatases PP1α and PP1γ induces a conformational change allowing for ATPase activity and enhanced affinity for viral dsRNA [206,207,208]. The ATPase activity of the helicase domain may increase the specificity of binding to viral dsRNA, instead of self-dsRNA that occurs during adenosine deaminase acting on RNA (ADAR1) dysfunction of dsRNA editing [206,209,210,211]. The ADAR1 enzyme edits mitochondrial self-RNAs by converting adenosine to inosine to prevent detection by MDA5 [211,212,213,214,215,216]. Mutations in *ADAR1* that result in decreased function and inappropriate detection of self-RNAs by MDA5 can cause Aicardi–Goutières syndrome, a Type I interferon-mediated auto-inflammatory neurological disease that occurs in the absence of infection [217,218]. Mutations in *ADAR1* and *IFIH1* may lead to hypersensitivity to self-RNA, resulting in aberrant IFN-α/β responses promoting AGS and T1D, respectively [215,219].

Once MDA5 has bound dsRNA, the CARD domains form a filament structure and mediate downstream signaling by interacting with mitochondrial antiviral signaling protein (MAVS). Interactions between CARD and MAVS leads to the phosphorylation of NF-κB, IRF-3, and IRF-7 that subsequently generate an antiviral response consisting of chemokines, cytokines, and IFN-stimulated genes [205,220,221,222,223]. CARD signaling is independent of helicase ATPase activity since IFN-β synthesis is not impaired in ATPase-deficient MDA5 protein [206]. There is a close interaction between the first helicase domain and the CTD, which provides specificity for binding of dsRNA for MDA5. Mutations in the CTD may modify the specificity of viral dsRNA binding and lead to non-functional or constitutively active MDA5 [205]. In the absence of the CTD, MDA5 remains in an inactive form with little to no capacity to bind dsRNA [224]. Further understanding of each functional domain of MDA5 and their interactions will enhance our knowledge of the role of *IFIH1* SNPs in T1D.

## 7. T1D Risk-Associated SNPs in *IFIH1*

Two SNPs in human *IFIH1* associated with risk for T1D [14,219,225,226] include SNPs rs1990760 (A946T) and rs3747517 (R843H) with odds ratios of 1.9 and 1.7, respectively [219,227,228] (Figure 2). SNP A946T is present at an allelic frequency of ≈0.36 and leads to a change in residue 946 within the CTD from a nonpolar alanine to a polar threonine [229,230]. This mutation in the CTD may alter protein structure and modify MDA5 specificity for long dsRNA from replicating enteroviruses and other RNA viruses resulting in increased dsRNA binding. Changing from a nonpolar residue to a polar residue may reduce the heat capacity change of the unfolded protein by 30%, making it kinetically easier for the protein to unfold into its active form for constitutive signaling [231].

The study of the SNP A946T has been fairly limited with clear discrepancies on its effect. Homozygous expression of the 946T variant in human PBMCs and HEK293T cells increased basal IFN-β synthesis following EMCV infection and transfection with poly(I:C) [219]. Mouse embryonic fibroblasts homozygous for the 946T variant had constitutive MDA5 activity but an impaired response to EMCV infection [230]. Conversely, heterozygous expression of the 946T variant in human PBMCs and β-cells results in increased IFN-β and MDA5 synthesis in response to naked poly(I:C) and CVB infection compared to diabetic patients homozygous for the 946T variant and healthy non-diabetic patients lacking the 946T variant [232,233]. The difference in antiviral responses to stimuli between these studies may be due to different interactions between CVB, transfected poly(I:C), naked poly(I:C), and EMCV with MDA5. The most consistent phenotype was observed when cells were infected with CVB, but more studies are required to fully understand the role of A946T expression in T1D development.

The second SNP associated with T1D risk R843H has been studied far less than the A946T SNP. R843H is present at an allelic frequency of ≈0.41 and leads to residue 843 changing from a positively charged arginine to a positively charged histidine [229]. This mutation is within the helicase and pincer domain near both ATP binding and phosphorylation sites [229,234]. It remains unclear how the R843H variant affects MDA5 function, but mutations near ATP binding and phosphorylation sites may alter viral sensing and IFN-α/β synthesis. Arginine is a strongly charged amino acid with a pKa_3_ of ≈13.8 at physiological pH and has a robust role in protein structure and folding [235]; whereas, histidine has a lower positive charge than arginine with a pKa_3_ ≈6.3 [236]. Charged residues are typically located in functional regions of proteins. Substituting in a weakly charged residue may alter the protein’s ability to bind dsRNA and the electrostatic interaction required for proper protein folding [237].

A pharmacogenomics study of patients with multiple sclerosis found that individuals with the R843H SNP were non-responsive to IFN-β therapy [238]. This study may indicate that the R843H SNP may impair response to IFN-β therapy due to high basal IFN levels. A meta-analysis looking at viral sensing genes within African populations discovered that the R843H SNP conferred an evolutionary advantage and may alter viral sensing [19]. The A946T and R843H SNPs may lead to a propensity for T1D, due to increased basal IFN synthesis, but more research is required to fully understand how these SNPs contribute to T1D development.

## 8. T1D Protection-Associated SNPs in *IFIH1*

Four SNPs in human *IFIH1* have been associated with protection from T1D development [225,226,239]. SNPs rs35744605 (E627x), rs35744605 (I923V), rs35337543 (*IFIH1*.intronic^Δ8^), and rs35732034 (*IFIH1*.intronic^Δ14^) are associated with T1D protection with odds ratios of 0.69, 0.51, 0.58, and 0.74, respectively [226,239,240,241] (Figure 2). These four SNPs are extremely rare within the human population. SNP E627x, I923V, *IFIH1*.intronic^Δ8^, and *IFIH1*.intronic^Δ14^ have allelic frequencies of ≈0.0013, ≈0.0024, ≈0.0056, and ≈0.0062, respectively [229]. The E627x SNP is a loss-of-function nonsense mutation that results in a premature stop codon at residue 627 and truncation of the protein in the helicase domain [239]. Mouse embryonic fibroblasts expressing the 627x variant completely lost dsRNA binding ability and were unable to upregulate IFN-β in response to poly(I:C) [239]. A cohort study on T1D PBMCs found that ≈1% of T1D patients express the E627x SNP, while ≈3% had the I923V SNP [240]. PBMCs expressing the 627x variant had reduced IFN-β synthesis following 1, 5, 15, and 36 h of poly(I:C) transfection compared to the E627 variant from diabetic individuals, regardless of prior enteroviral infection [240].

The E627x SNP is extremely rare and only heterozygous expression has been identified within the human population [240,241]. To date no transgenic mice with E627 SNP have been made; however, NOD mice heterozygous for *Ifih1* (MDA5^+/−^) can be used to further understand the consequences of reduced MDA5 expression on T1D. NOD.MDA5^+/−^ exhibit a delay in spontaneous and CVB-induced T1D, have reduced CD4 and CD8 T cell effector responses, and have enhanced Treg functionality compared to WT NOD mice [242]. Overall, the E627x SNP may lead to reduced effector T cells and enhanced Treg functionality, providing protection from T1D, as observed with the MDA5^+/−^ mouse model.

A second loss-of-function SNP in *IFIH1*, I923V, results in residue 923 changing from a non-polar isoleucine to a non-polar valine within the CTD. This mutation is only four residues away from H927 which plays a role in dsRNA binding [239,240]. Interestingly, the I923V SNP does not affect dsRNA binding or overall protein expression but does lead to reduced ATP hydrolysis and IFN-β synthesis after poly(I:C) transfection [239,240]. HEK293T cells transfected with the 923V variant had reduced IFN-β synthesis at baseline and after EMCV infection [219]. The 923V variant is less efficient at filament formation and dissociates from bound dsRNA four times faster than the I923 variant of MDA5 [243]. The dysregulated filament kinetics of MDA5 containing the 923V variant might reduce signaling, leading to reduced basal and virus-induced IFN synthesis, and elicit a delay in T1D.

Two intronic splice variants have also been associated with T1D, *IFIH1*.intronic^Δ8^ and *IFIH1*.intronic ^Δ14^, expression can be homozygous or heterozygous in individuals [226,241,244]. The *IFIH1*.intronic^Δ8^ SNP results in a deletion of exon 8 and 39 residues within the end of the helicase 1 domain and the linker region between helicase domains 1 and 2 but does not cause a frameshift. The second intronic SNP *IFIH1*.intronic^Δ14^ is a splice variant that causes skipping of exon 14, which results in a frameshift and premature generation of a stop codon in exon 15 [244]. The subsequent MDA5 protein lacks 153 residues and loses the CTD domain. Individuals heterozygous for *IFIH1*.intronic^Δ8^ and *IFIH1*.intronic^Δ14^ have reduced mRNA transcription of *IFIH1* [241], and expression of *IFIH1*.intronic^Δ8^ and *IFIH1*.intronic^Δ14^ in 293T cells have reduced IFN-β synthesis and ATP hydrolysis [244]. Therefore, reduced *IFIH1* mRNA levels may decrease MDA5 protein, resulting in dampened antiviral signaling and proinflammatory responses. *IFIH1*.intronic^Δ8^ and *IFIH1*.intronic^Δ14^ lose a portion of the helicase 1 domain and the CTD, respectively, this should impair dsRNA binding or increase dsRNA dissociation. Unfortunately, no dsRNA binding studies have yet been performed on *IFIH1*.intronic^Δ8^ or *IFIH1*.intronic^Δ14^. Overall, SNPs E627x, I923V, *IFIH1*.intronic^Δ8^, and *IFIH1*.intronic^Δ14^ either cause loss-of-function or reduced expression of MDA5 resulting in associated T1D protection due to decreased basal and viral-induced IFN-α/β synthesis.

## 9. Conclusions

The increased prevalence of T1D worldwide is a significant healthcare concern, but our understanding of how genetic risk factors synergize with environmental triggers to exacerbate autoimmunity is not well defined. One mechanism may be due to exacerbated MDA5-dependent antiviral responses following CVB infection of genetically-susceptible individuals for T1D. T1D risk- and protection-associated SNPs in *IFIH1* are present in the helicase, pincer, and CTD regions of MDA5 causing dysregulated IFNα/β synthesis and dsRNA binding. However, it remains unclear how these SNPs alter MDA5 structure and other unknown functions leading to T1D risk or protection. Mutations in *IFIH1* that increase basal or virus-induced IFN-α/β synthesis may cause aberrant immune cell activation and chronic inflammation, which could lead to destabilization of immunosuppressive Tregs and bystander activation of autoreactive CD4 and CD8 T cells to induce T1D. Conversely, mutations in *IFIH1* that lead to loss-of-function and reduced expression of MDA5 can potentially provide protection from T1D by reducing basal and virus-induced IFN-α/β synthesis. Therefore, the hyperinflammatory response to enteroviral infections may be a key initiator of T1D. MDA5-dependent responses can dictate whether T1D progression or protection ensues. Unfortunately, there is little mechanistic data elucidating the interaction between MDA5, CVB, and T1D to fully grasp how genetics and environment work together to induce T1D. Understanding these interactions may result in the development of novel therapies to prevent T1D, such as vaccines, antiviral medications, or MDA5 small molecule inhibitors that reduce but do not impair MDA5 function.
